# Polarization Spin Inversion with Nonlinear Plasmon Scattering

**DOI:** 10.1021/acsomega.4c09135

**Published:** 2025-01-28

**Authors:** Pritam Khan, Grace Brennan, Syed A. M. Tofail, Ning Liu, Christophe Silien

**Affiliations:** Department of Physics and Bernal Institute, University of Limerick, Castletroy, Co., Limerick V94 T9PX, Ireland

## Abstract

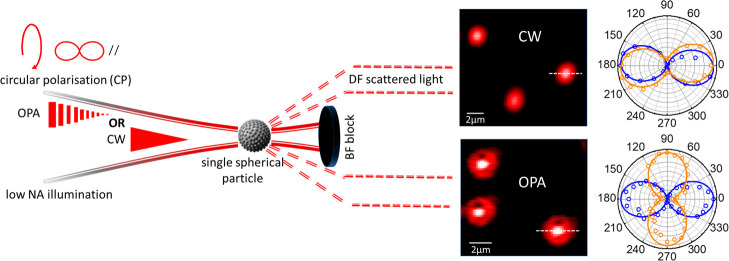

We report on circularly polarized Gaussian beam spin angular momenta that can be inverted upon scattering with quadrupole plasmon modes. The conditions for such conversion are met with high-angle collection, dark-field scattering microscopy on spherical plasmonic particles. We further report that silvered nanoporous silica microparticles exhibit a strong nonlinearity in their scattering, specifically a reverse saturated scattering (RSS), when exposed to high laser power densities on the sample of ca. 5 GW/cm^2^. Handedness conversion by these microparticles is only observed at wavelengths tuned to the quadrupole modes. Conversely, the scattering remains linear, and the handedness is unchanged, when the same particles are illuminated with low laser power densities of ca. 10 W/cm^2^. We infer that RSS tuned to the quadrupole modes sufficiently enhances their contribution so that they dominate the high-angle scattering, thereby justifying the light spin inversion. Moreover, the addition of a self-assembled monolayer of ethynylaniline (EA) on the microparticles results in handedness conversion for both low and high incident power, as expected from preferable dipole damping and plasmon mode red shift. This demonstrates that optical nonlinearity in scattering can be exploited for polarization tuning in plasmonic metamaterials.

## Introduction

Light-matter interactions in metal nanoparticles and metamaterial nanostructures lead to the excitation of surface plasmon resonances that provide efficient extinction (absorption and scattering) of incident light and find applications in optics and optoelectronics.^1−5^ Surface plasmon resonances at visible and near-infrared wavelengths with gold^6^ and silver^7^ are particularly important thanks to favorable optical confinement that results in a huge enhancement of the local electromagnetic field. For spherical particles with size smaller than the wavelength of the incident light, the overall optical response is largely dominated by dipolar scattering.^8,9^ The contribution of higher-order multipole plasmon modes (e.g., quadrupoles and octupoles) to the scattering is measured in specific experimental geometry and aspherical particles.^8−11^ The multipolar response is also strongly influenced by plasmon damping, for example arising from the adsorption of self-assembled monolayers of molecules on the particles. Dipolar plasmon modes show higher damping than quadrupolar modes and the latter can dominate the scattering after molecular adsorption in aspherical particles.^12,13^ Quadrupole modes are found at shorter wavelengths than dipoles and also have narrower absorption peak.^9,14,15^ It results that quadrupoles can provide enhanced spectroscopic signal of nearby molecules or semiconductor particles^16^, yield larger figures of merit for refractive index sensing,^13,17^ and exhibit larger surface-enhanced Raman scattering signal.^18,19^

Since localized surface plasmon resonances increase the electromagnetic field, gold and silver nanoparticles exhibit strong nonlinear responses.^20,21^ For example, the nonlinear Kerr coefficient for gold nanoparticles is ca. 10^–8^ esu, which is 6 orders of magnitude stronger compared to fused silica^22^. Applications of optical nonlinearity in plasmons include optical switching^23,24^ and limiting^25^, all-optical signal processing^26^, and super-resolution microscopy^23^. Nonlinearities in plasmonic nanoparticles include saturable absorption and saturable scattering (SS).^20,21,27^ These have been reported for 80–100 nm Au particles with continuous wave (CW) power densities of 10^5^–10^6^ W/cm^2^. Reverse saturable absorption and scattering (RSS) were also reported for CW power densities above these values.^20,21,28^ With optical scanning microscopy, when the optical scattering is recorded, SS produces images where single particles exhibit a dip or plateau at their center (instead of the nominal Gaussian profile seen with linear scattering), while RSS produces images where single particles exhibit enhanced scattering at their center.^20,21,29^ Other nonlinear optical responses such as absorption also induce similar reshaping of the otherwise Gaussian spatial profiles^30^ and the process has been called x-scan^29^ in reference to the more established z-scan technique.^31−33^ Optical nonlinearity in Au nanoparticles has also been reported with 100 fs pulsed lasers with a peak power of ca. 5 GW/cm^2^.^34^

In a recent work^35^, we discussed that when metallic nanoparticles are illuminated with a circularly polarized Gaussian beam and that quadrupole modes dominate the measured scattering, the handedness is inverted. Indeed, quadrupole modes possess the necessary orbital angular momentum that upon coupling, leads to inversion of the spin angular momentum of the incident light. This has been measured by high-angle collection, dark-field (DF) scattering microscopy for 300 nm Au nanoparticles across the visible spectrum and for Ag nanoparticles-modified nanoporous 1.5 μm silica microparticles (n-SiO_2_@Ag) at short visible wavelengths. Interestingly, for these microparticles, after the adsorption of a self-assembled monolayers of short organic molecules on the silver, the inversion is seen at both short and long visible wavelengths. The latter was explained from the quadrupole scattering cross-section being dampened to a lesser extent than the dipole, upon molecular adsorption^36^.

In this paper, we further discuss that nonlinearity in the plasmon scattering is also observed for n-SiO_2_@Ag microparticles and that it can be used to modify the relative contribution of quadrupole and dipole modes, and in turn modify the handedness of a scattered circularly polarized light. These n-SiO_2_@Ag microparticles are thus reinvestigated by high-angle collection DF microscopy with circularly polarized laser illumination both at low power density (ca. 10 W/cm^2^) with a CW laser and at high power density (ca. 5 GW/cm^2^) with a pulsed laser. These power densities reveal linear and nonlinear (RSS) scattering, respectively. We observe that incident and scattered handedness are opposite only with RSS and with the wavelength tuned toward the quadrupole modes (i.e., 540 nm) and thus explain the conversion from a relative increase in the quadrupolar contribution arising with RSS. This demonstrates that optical nonlinearity in scattering can be exploited for polarization control in plasmonic metamaterials. The addition of a self-assembled monolayer of ethynylaniline (EA), that was found earlier to preferably dampen dipole over quadrupole modes, results in handedness conversion for both low and high incident power densities. Thus, with EA, the nonlinearity in the scattering is not necessary for conversion. These results highlight straightforward molecular detection at single particle level using DF microscopy, low-cost CW lasers, and isotropic spherical microparticles such as n-SiO_2_@Ag that were found to be highly reproducible and simple to prepare^37^.

## Results and Discussion

**Figure 1:**
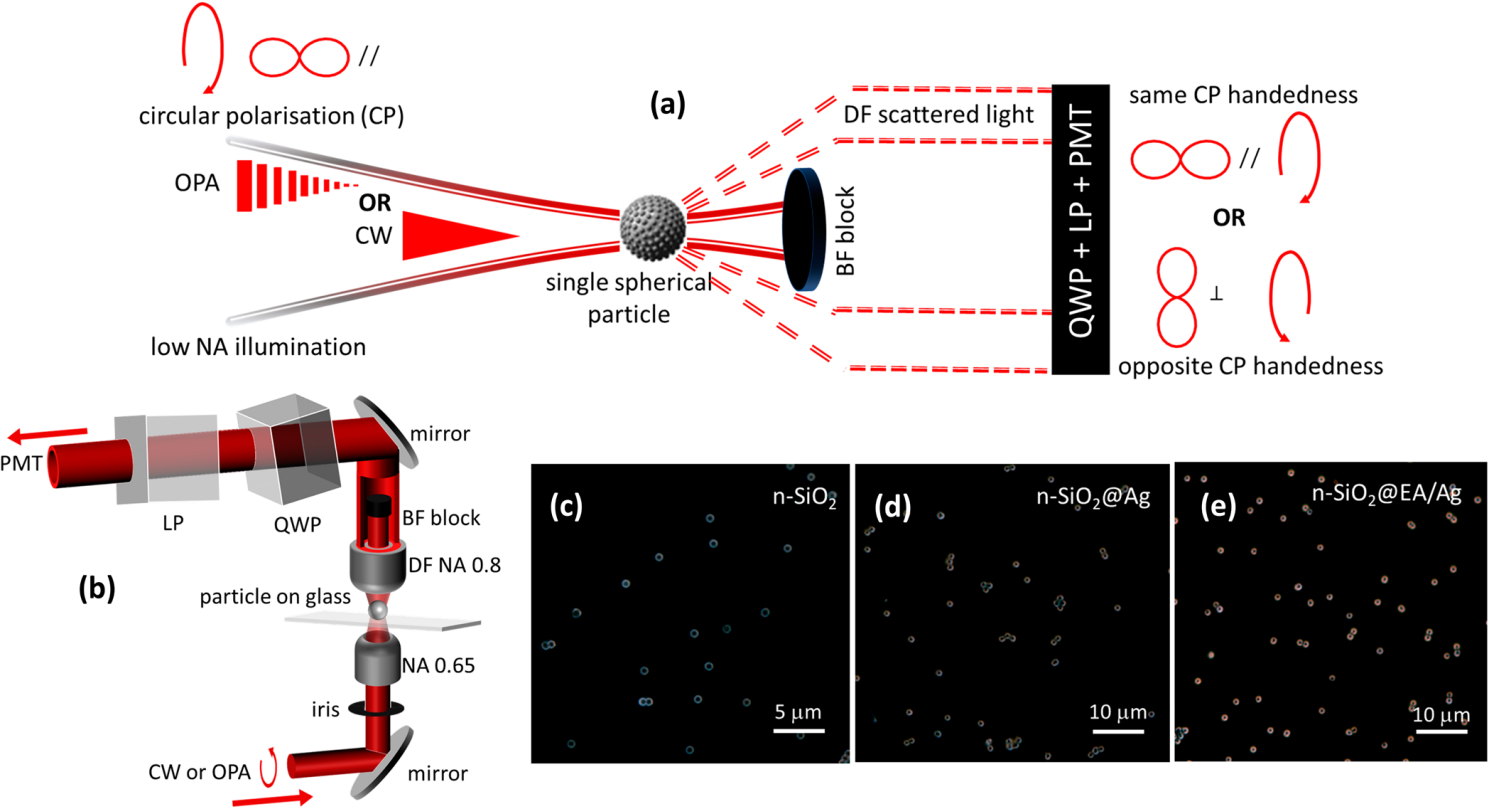
(a) Schematic of the experiment where a single particle is illuminated with a low NA focused circularly polarized Gaussian laser (OPA or CW) and the scattered light is collected at high angle in the DF, with the BF blocked, with a QWP-LP pair, and with a PMT. The QWP is adjusted so that when the collimated scattered circular polarization (CP) has the same or opposite handedness as the incident light, the polar plot is at 0° (//) or 90° (⊥), respectively. (b) Diagram of the DF transmission microscope with controllable input iris, removable low-NA beam (BF) block, wavelength tunable QWP, and LP analyzer on 360° rotation mount. (c,d,e) DF microscopy images of n-SiO_2_, n-SiO_2_@Ag, and n-SiO_2_@EA/Ag microparticles recorded in reflection, with high NA DF illumination, and a white light source.

Figure 1(a) depicts the high-angle collection, DF polarimetric microscopy that was setup for this study, with the illumination achieved using either a 3 ps pulsed optical parametric amplifier (OPA) (5 GW/cm^2^ at sample focus) or a CW laser (10 W/cm^2^). In both cases, the incident beams are made circularly polarized and produce Gaussian focuses on the sample (see below). The illumination is achieved using a 0.6 NA objective and the scattered light is measured with a photomultiplier tube (PMT) after collection with a 0.8 NA DF objective and analysis by a combination of wavelength-tunable quarter-wave plate (QWP) and 360° rotatable linear polarizer (LP) (details in METHODS and Figure 1(b)). A removable, low-NA-beam block is used to record DF polarization-resolved images. The QWP and LP are aligned in such a way that when scattered and incident circularly polarized lights have same handedness, the polar plot is oriented at 0° (//) and when they are of opposite handedness, the polar plot is with its main axis at 90° (⊥). In the experiments, we recorded images at various LP angles (with the QWP in place) and reconstructed polar plots for individual particles afterward. The samples analyzed were prepared with dispersed nanoporous silica microparticles (ca. diameter 1.5 μm, 92 Å average pore diameter, n-SiO_2_, Glantreo) drop-casted on glass microscopy slides, the same microparticles after reduction of Ag nanoparticles in the nanopores (n-SiO_2_@Ag) and also after further adsorption of EA on the silver (n-SiO_2_@EA/Ag). The preparation and characterization of these microparticles was reported earlier^37^.

Reflection, white light DF images recorded using a Zeiss Axiovision (equipped with a 20× Zeiss Epiplan 0.4 NA objective) are shown in Figure 1(c)-(e). These images confirm that single microparticles and small islands can be readily identified. n-SiO_2_@EA/Ag microparticles appear redder than n-SiO_2_@Ag. This is consistent with the plasmon scattering band shifting toward longer wavelengths from ca. 704 to 755 nm after the adsorption of EA, that we measured with a 0.9 NA brightfield (BF) collection objective (visible broadband illumination with a F-type oil immersion 1.2–1.4 NA DF condenser) (see Figure S1, Supporting Information (SI)). Since the particles are spherical, these spectral bands are dominated by dipole scattering. Quadrupole modes are typically found at wavelengths that are in average 25% shorter than dipole modes (see Table S1, Supporting Information) but are not expected to significantly contribute upon BF collection geometry^8^. However, the asymmetry in the main scattering peak seen in BF collection below ca. 600 nm, along with the relative increase in scattering in DF collection at the same wavelengths (see Figure S1, Supporting Information) are reminiscent of quadrupole modes.

**Figure 2:**
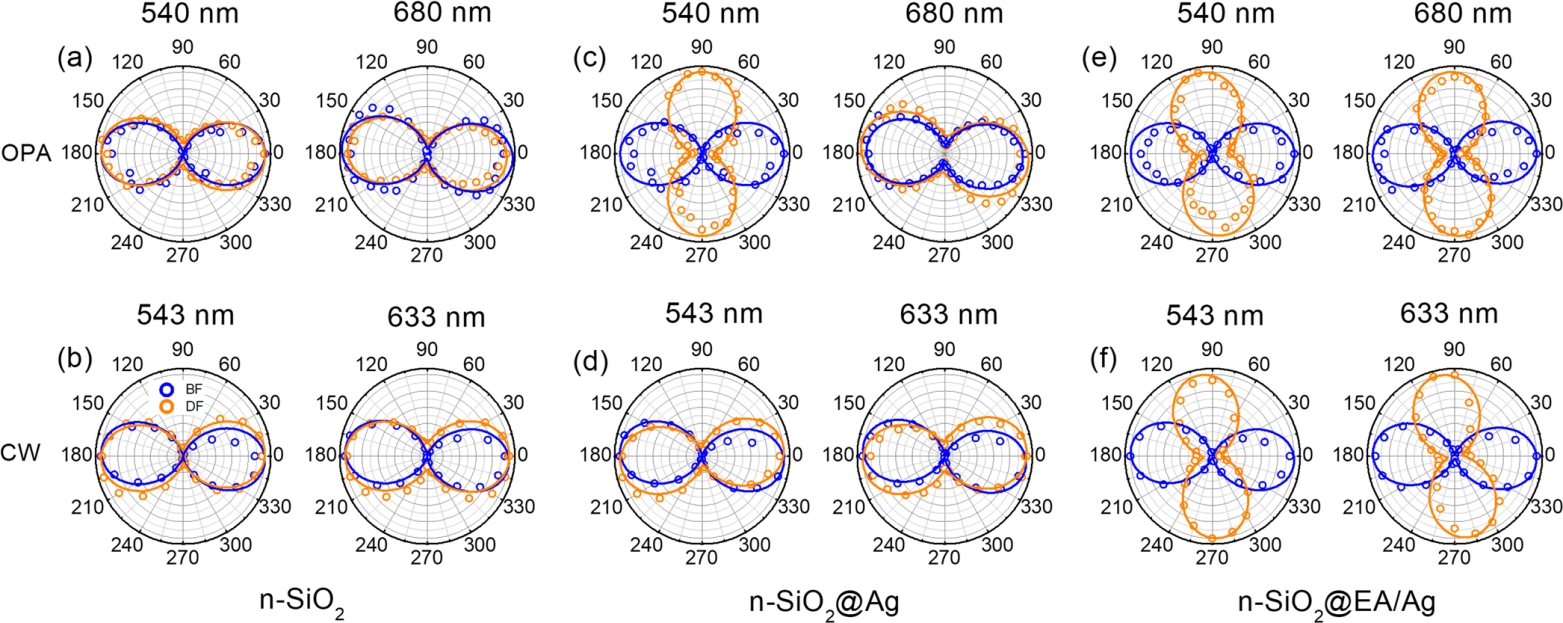
(a,b) Normalized single-particle polar plots (BF in blue and DF in orange) with circular polarization incidence and QWP-LP detection, for the OPA tuned at 540 and 680 nm (top row) and two CW lasers at 543 and 633 nm (bottom row), for single n-SiO_2_ microspheres. (c,d) Same for n-SiO_2_@Ag. (e,f) Same for n-SiO_2_@EA/Ag.

Figure 2 shows normalized single-particle polar plots in both BF and DF with the setup described in Figure 1(b). For low power density irradiation, we used 543 and 633 nm CW lasers. For high power density irradiation, we used the OPA tuned at 540 and 680 nm. In our experiments, the BF data (see blue data in Figure 2) measure the transmitted beam and the observation of well-defined // polarization in BF confirms the quality of the beam alignment and circular polarization for both OPA and CW illuminations. We also checked that without the QWP, the BF polar plots are in good agreement with a circle confirming the incident circular polarization (Figure S2, Supporting Information).

In DF, where the scattering dominates, for n-SiO_2_ (see orange data in Figure 2(a) and (b)) we do not observe any remarkable change in the polarization with either wavelength and either laser (see also Figure S3, Supporting Information). Therefore, we established n-SiO_2_ microparticles as reference material for the // circular polarization, in agreement with our earlier report^20^. For n-SiO_2_@Ag, with the OPA (see Figure 2(c)), the handedness of the scattered light in the DF is inverted at 540 nm, while the inversion is not observed at 680 nm. In contrast to what is measured with the OPA, the handedness is not inverted neither with green nor red CW lasers (see Figure 2(d)). DF images of n-SiO_2_@Ag with CW and OPA are shown in Figure S4, Supporting Information. We argue below that the differences between OPA and CW data stem from the nonlinear plasmonic scattering at sufficiently high power densities on the samples.

In Figure 2(e) and (f), we show the polar plots after addition of EA on the silvered microparticles for OPA and CW lasers, respectively. For these n-SiO_2_@EA/Ag particles, all the DF polar plots exhibit conversion of handedness, with the ⊥ scattering clearly dominating for all wavelengths and lasers (see also Figure S5, Supporting Information). Those observations are interesting with respect to potential applications since CW diode lasers are indeed more accessible, compact, and cost-effective. Those observations also confirm that the nonlinearity in the scattering that is expected with the OPA (and that will be established below) is not the root cause of the circular polarization inversion although it does clearly facilitate it. Indeed, we established earlier that the inversion stems from an increased relative quadrupolar contribution in the collected scattering^20^.

**Figure 3:**
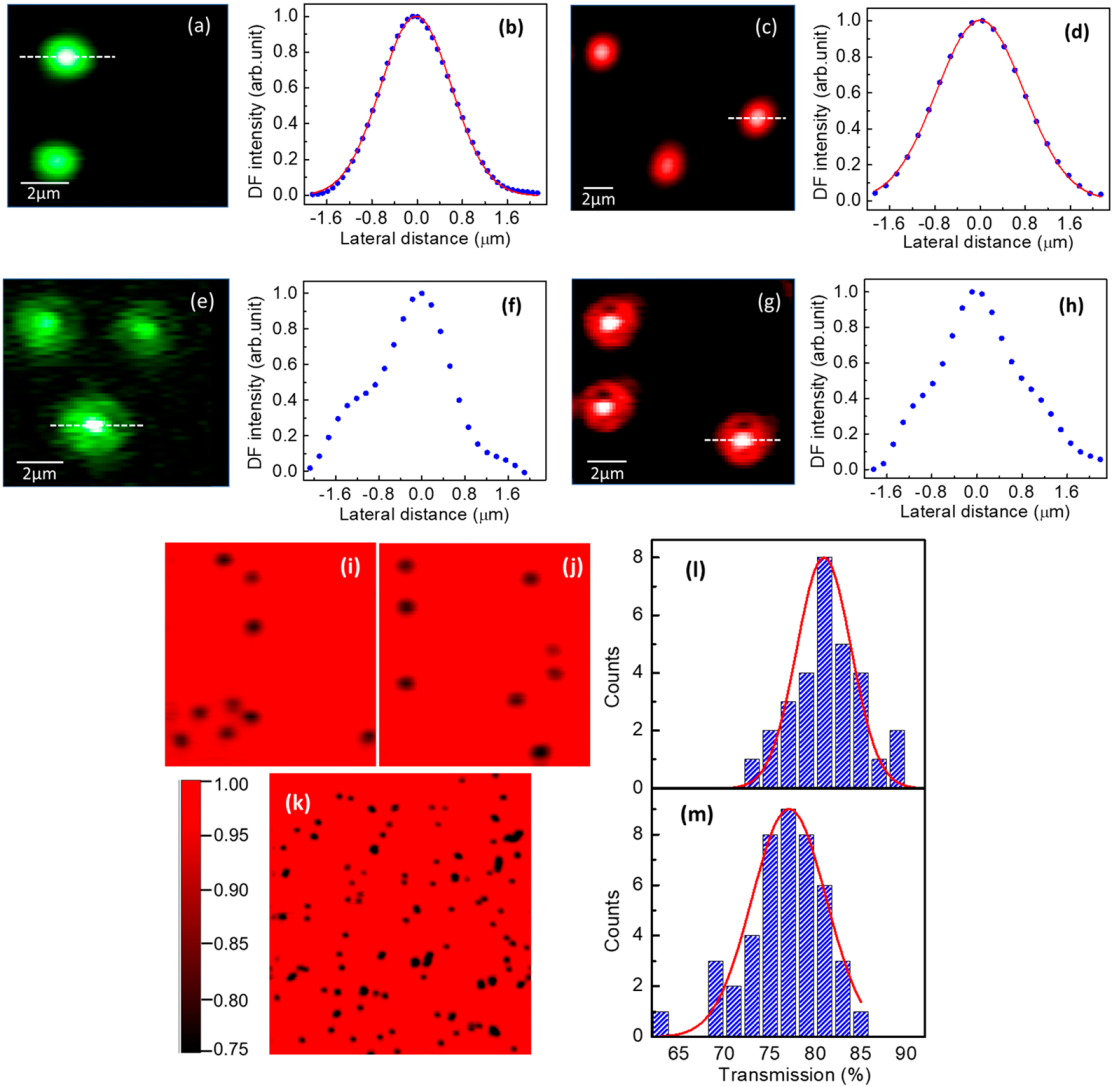
(a) DF image of n-SiO_2_@Ag microparticles with a 543 nm CW laser. (b) Line profile extracted from (a) with Gaussian fit in red. (c,d) Same with a 633 nm CW laser. (e,f) Same with the OPA tuned at 540 nm. The deviation from a Gaussian profile highlights RSS. (g,h) Same with the OPA tuned at 680 nm. (i,j) BF transmission images of n-SiO_2_@Ag microparticles with the 633 nm CW laser (horizontal scale 50 μm). (k) Same with the OPA at 680 nm (horizontal scale 100 μm). (l,m) Histograms of the percentage of light measured in bright field transmission when the beam is at the center of single n-SiO_2_@Ag microparticles (with respect to when the beam is away from any particles) with the 633 nm CW and the OPA at 680 nm, respectively. Gaussian fits are shown in red.

As presented above in this manuscript, optical nonlinearity affects how particles are imaged.^22,23,31^ In Figure 3, we present DF and BF high resolution images of n-SiO_2_@Ag particles and line profiles, recorded with both OPA and CW lasers. Complementary images and line profiles for n-SiO_2_ are presented in Figure S6, Supporting Information. We note that the images of single particles and line profiles for n-SiO_2_ particles are well fitted with Gaussian functions for both CW and OPA (Figure S6(a)-(d), Supporting Information), which confirms that the imaging point-spread functions in both cases are similar and mostly determined by the optics in the microscope. In addition, this also confirms the absence of optical nonlinearity where there is no silver and thus no plasmon. For the n-SiO_2_@Ag microparticles, with the CW lasers (Figure 3(a)-(d)), Gaussian images and line profiles are also recorded and in line with the n-SiO_2_ microparticles, there is thus no evidence of optical nonlinearity. This is consistent with the power density (ca. 10 W/cm^2^) that is well below the values of 10^5^–10^6^ W/cm^2^ reported by others to observe SS or RSS.^22,23,38^ By contrast, when these same n-SiO_2_@Ag microparticles are imaged with the OPA (Figure 3(e)-(h)), DF images and line profiles reveal a remarkable deviation from the original Gaussian, with the unambiguous appearance of a bright center. Those observations are consistent with RSS and indeed the power density with the OPA here (ca. 5 GW/cm^2^) is well above the values where nonlinearity was reported in other plasmonic particles.^22,23,38^ Moreover, for n-SiO_2_@EA/Ag microparticles, the DF images (see Figure S6(h), Supporting Information) also reveal the same deviation from Gaussian profiles with OPA illumination. Thus, altogether, RSS is systematically observed with the high power density OPA, in all the cases where the Ag nanoparticles were reduced on the microparticles, and thus as long as there are plasmons.

RSS is expected to be accompanied by a reduced transmission in BF since a larger fraction of the incident light is then scattered. We present BF images of n-SiO_2_@Ag microparticles recorded with the red CW laser in Figure 3(i) and (j), and with the red OPA in Figure 3(k). We measured line profiles across several (30 for CW and 44 for OPA) particles (for examples of line profiles, see Figure S7, Supporting Information) and present histograms of the loss in transmitted power at the center of the particles (with respect to the transparent background of the glass substrates) in Figures 3(l) and (m), for the red CW and OPA respectively. It is found that when the focus is centered on a n-SiO_2_@Ag microparticle, the transmitted intensity is statistically reduced to 82% with the CW laser and to 77% with the OPA. Thus, the BF transmission images also support our proposition of RSS with the OPA.

We have argued earlier that the circular polarization conversion or spin inversion, measured in high-angle collection DF microscopy on spherical plasmonic particles, matches experimental conditions in which the quadrupole modes dominate the scattering^20^. Here, for the n-SiO_2_@Ag, we reported in Figure 2 that such inversion is indeed observed only where RSS occurs (i.e., OPA), and only at 540 nm and not at 680 nm. The spectrum in Figure S1, Supporting Information revealed that the dipolar band is centered in the red at ca. 704 nm, and the data in Table S1, Supporting Information indicate that quadrupole modes are statistically with a wavelength 25% shorter than dipoles. For n-SiO_2_@Ag, the quadrupole resonance is thus expected in the green, at ca. 528 nm. Since others reported that RSS is a wavelength-dependent mechanism that is not observed when the wavelength is set away from the plasmon mode of interest and that occurs at power density thresholds that are minimum when at resonance,^20,21,38^ we propose that RSS provides here a mechanism that relatively enhances the quadrupolar contributions and thus the circular polarization conversion when the OPA is tuned in the green (in comparison to the linear regime, probed with the CW lasers). Accordingly, we can argue that when the OPA is tuned in the red, the relative enhancement of the dipolar contributions only strengthens the incident handedness, since the dipole modes do not carry the orbital angular momentum required for the circular polarization conversion.

The adsorption of EA has an interestingly large effect in our experiment, since for n-SiO_2_@EA/Ag microparticles, both CW and OPA illumination exhibit handedness inversion across the entire visible range. The model in ref [^20^] relates handedness inversion to quadrupole-dominated scattering and we note here that two EA-driven changes support indeed quadrupole scattering against dipole within the visible wavelengths under study. First, the overall scattering profile red shifts upon addition of EA (see Figure S1, Supporting Information) and thus the relative contribution of the dipole modes decreases. Second, differences in damping between dipole and quadrupole plasmon modes have been reported in other works. For examples, for silica-gold core–shell nanoparticles, a relative increase of the quadrupoles (against dipoles) was measured when the refractive index of the embedding medium is increased^12^, and for Au nanoprisms, where the extinction spectra reveal that dipolar resonances exhibit higher damping versus quadrupole ones upon adsorption of thiol-based molecules^21^. Assuming the same damping applies here, the quadrupole contribution to the scattering is also reinforced for SiO_2_@EA/Ag.

## Conclusions

We demonstrated that plasmonic metamaterial particles such as n-SiO_2_@Ag exhibit a nonlinear response (RSS) when irradiated at high power densities (ca. 5 GW/cm^2^). When the wavelength is tuned toward the quadrupolar plasmonic modes, we further confirm a conversion of the scattered circular polarization handedness, measured with high-angle collection DF microscopy. These two observations are rationalized with RSS selectively enhancing the quadrupolar contribution to the scattering and with the understanding that these modes exhibit indeed the orbital angular moment required for spin inversion of the input beam.^20,39−41^ The implication is that our measurements reveal a very strong coupling between scattering polarization and nonlinearity in the plasmon.

Notably, even at power densities as low as 10 W/cm^2^, the absorption of EA on the microspheres leads to a same conversion of the handedness. It is remarkable that EA-induced damping and wavelength-tuned RSS both act at reinforcing quadrupolar contribution (seen here from the scattered circular polarization handedness conversion). Metamaterials applications (e.g., optical sensors, switch, and limiter) relying on polarization and on spin angular momentum inversion can benefit from these complementary parameters.

## Methods

We built an inverted transmission DF microscope in which a long working distance low NA objective (50× Leitz PLAN L, NA = 0.60) was used for illumination and a high NA DF objective (50× Olympus UMPlanFl, NA = 0.8) was used for collection (Figure 1(b)). A block was placed at the center of the back aperture to eliminate the forward BF transmission from the detection during DF imaging. A PMT (Hamamatsu, E717–500) was employed to measure the intensities in BF and DF. Incident circular polarization was achieved by inserting a wavelength-tunable QWP on the linearly polarized CW and OPA beams, before entering the DF microscope. A second QWP was placed at the detection before the PMT. The QWP was paired with a 360° rotatable linear polarizer and used in tandem to produce the experimental polar plots of the scattered intensities. We verified the incident circular polarization (Figure S2, SI) and QWP alignment in BF by focusing the beam away from any particles. To do so, we recorded a uniform intensity for all linear polarizer angles in the absence of detection QWP and then confirmed polar plots of linear polarization in the presence of detection QWP, for all angles, by keeping the dominant polarization axis aligned with that of the incident laser linear polarization (i.e., // or 0°). The sample was mounted on a piezo-scanner (Physics Instruments) for focusing and scanning. The OPA (1 kHz, pulse duration ca. 3 ps) was a TOPAS from Light Conversion pumped by a Ti-Sapphire regenerative amplifier from Coherent and the CW lasers at 543 (5 mW) and 633 (4 mW) nm were from Melles Griot and Uniphase, respectively.

## Supporting Information Available

Figure S1: BF and DF spectra of nSiO_2_@Ag and nSiO_2_@EA/Ag; Figure S2: polar plots away from particles to confirm incident circular polarization; Figures S3–S5: DF images for green and red wavelengths, CW and OPA, crossed and parallel polarizations for n-SiO_2_, n-SiO_2_@Ag, and n-SiO_2_@EA/Ag; Figure S6: DF images and line profiles for n-SiO_2_ and n-SiO_2_@EA/Ag with CW and OPA; Figure S7: BF transmission line profiles for n-SiO_2_@Ag with CW and OPA; Table S1: list of some dipole and quadrupole band wavelengths reported in the literature (PDF)
