# Supplementary material for: Polarization Spin Inversion with Nonlinear Plasmon Scattering
Source: ACS Omega. 2025 Jan 28;10(5):4607–13. doi: 10.1021/acsomega.4c09135 (PMC11822691; doi:10.1021/acsomega.4c09135)
Supplement: Supplementary file 1 — ao4c09135_si_001.pdf [file ao4c09135_si_001.pdf]

Polarization spin inversion with non-linear plasmon scattering

Pritam Khan[1], Grace Brennan[2], Syed A. M. Tofail, Ning Liu*, and Christophe Silien*

Department of Physics and Bernal Institute, University of Limerick, Castletroy, Co. Limerick, V94 T9PX, Ireland

* *Ning.Liu@ul.ie* *and* *Christophe.Silien@ul.ie*

Supplementary figures and table

[1] Present address: Seagate Technology, Springtown Industrial Estate, BT48 0BF, Londonderry, UK.
[2] Present address: Microsoft Research, Cambridge Science park, Milton Rd, CB1 2FB, Cambridge, UK.

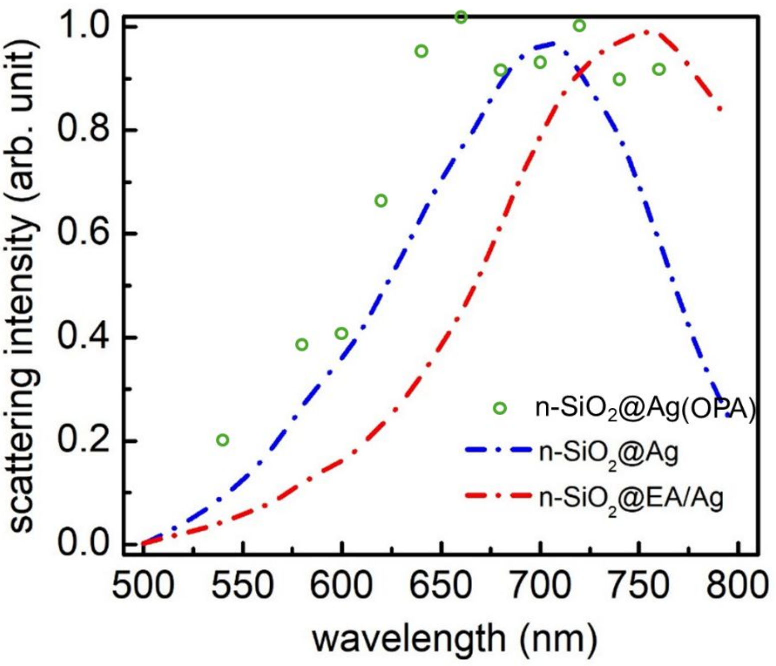

Figure S1. Dash-dot-dash lines: Averaged Dark-field scattering spectra of n-SiO_2@Ag (14 particles) and n-SiO_2@EA/Ag (10 particles) illuminated with a white light and measured with a spectrograph (Acton, Princeton Instruments). The spectra were corrected for the white light spectrum and normalized to 1. The scattering was collected with a brightfield 0.9 NA collection objective, and the illumination was with a F-type oil immersion 1.2-1.4 NA darkfield condenser. Green circles scatter plots: Averaged Dark-field scattering spectra of n-SiO_2@Ag (3 particles) recorded with OPA, low NA illumination and dark-field collection (polarization scheme not in place).

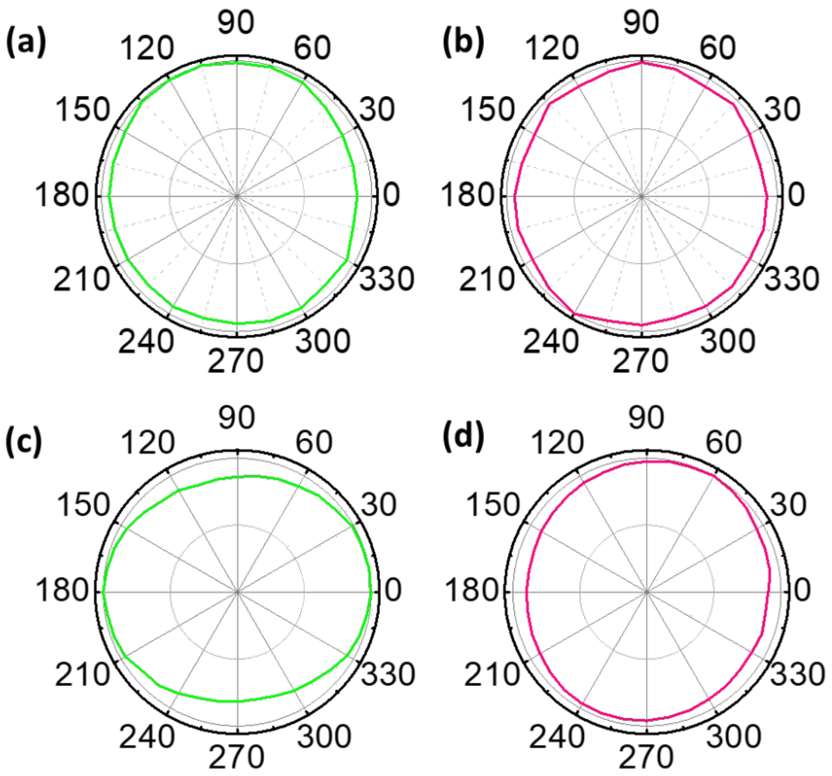

Figure S2. Polar plots measured with the system of Figure 1b but without the QWP, and away from any particles. These polar plots confirm the beams are circularly polarized. (a) 543 nm CW laser. (b) 633 nm CW laser. (c) OPA tuned at 540 nm. (d) OPA tuned at 680 nm.

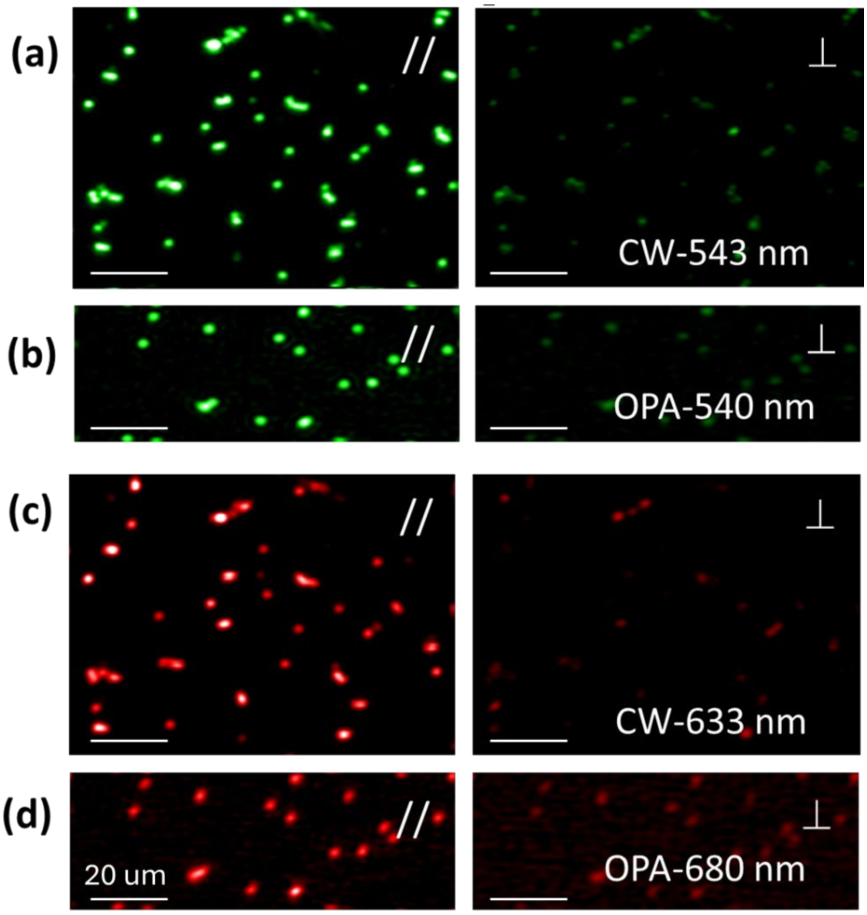

Figure S3. DF images of n-SiO_2 microspheres with (a) 543 nm CW laser, (b) OPA tuned at 540 nm, (c) 633 nm CW laser, and (d) OPA tuned at 680 nm. The images were recorded with the QWP-LP pair set for measuring the parallel (//) and perpendicular (⊥) components (left and right column, respectively). (All images horizontal size = 100 µm; scalebar = 20 µm; each // and ⊥ pairs are presented with same colour scheme).

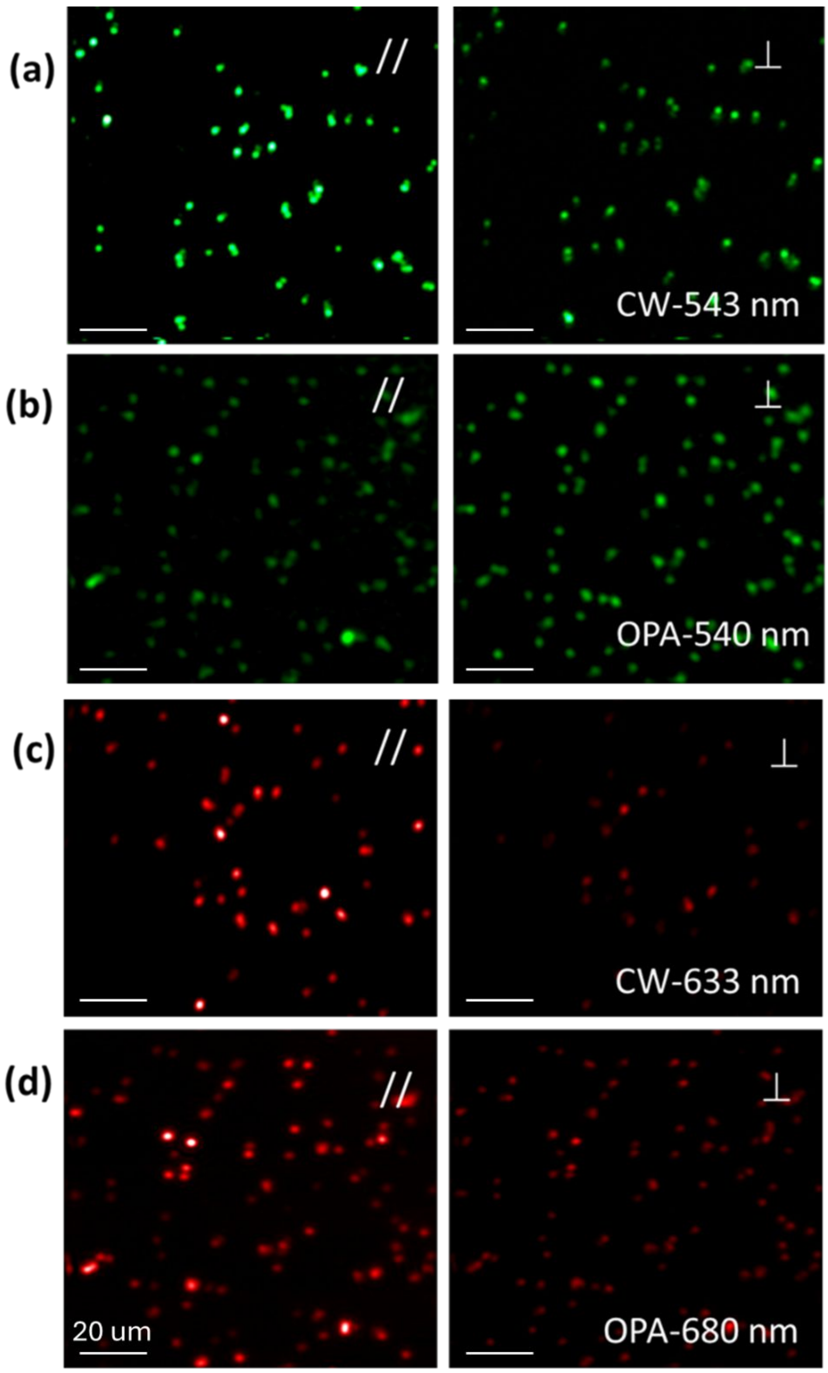

Figure S4. DF images of n-SiO_2@Ag microspheres with (a) 543 nm CW laser, (b) OPA tuned at 540 nm, (c) 633 nm CW laser, and (d) OPA tuned at 680 nm. The images were recorded with the QWP-LP pair set for measuring the parallel (//) and perpendicular (⊥) components (left and right column, respectively). (All images horizontal size = 110 µm; scalebar = 20 µm; each // and ⊥ pairs are presented with same colour scheme).

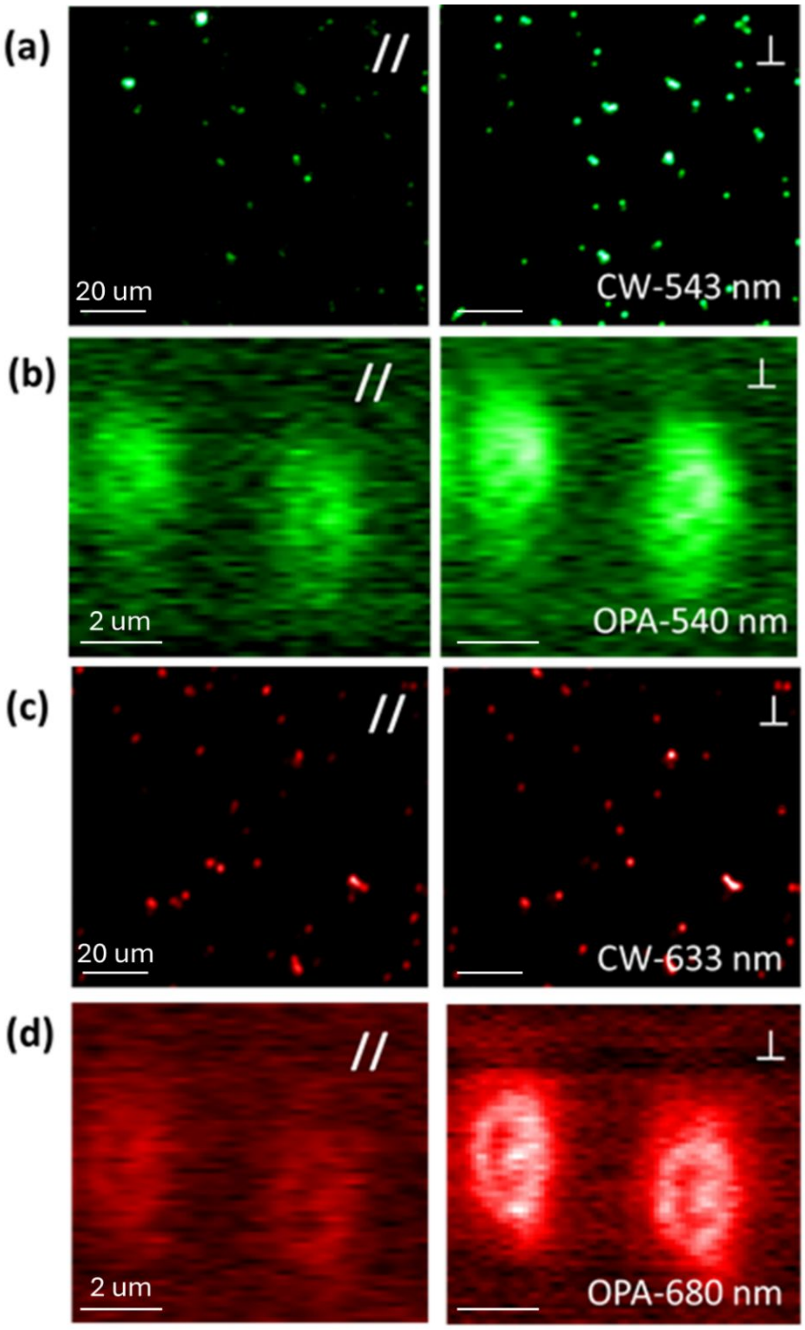

Figure S5. DF images of n-SiO_2@EA/Ag microspheres with (a) 543 nm CW laser, (b) OPA tuned at 540 nm, (c) 633 nm CW laser, and (d) OPA tuned at 680 nm. The images were recorded with the QWP-LP pair set for measuring the parallel (//) and perpendicular (⊥) components (left and right column, respectively). (For (a)-(c) horizontal size = 110 μm (scalebar = 20 μm) and for (b)-(d) horizontal size = 8.5 μm (scalebar = 2 μm), each // and ⊥ pairs are presented with same colour scheme).

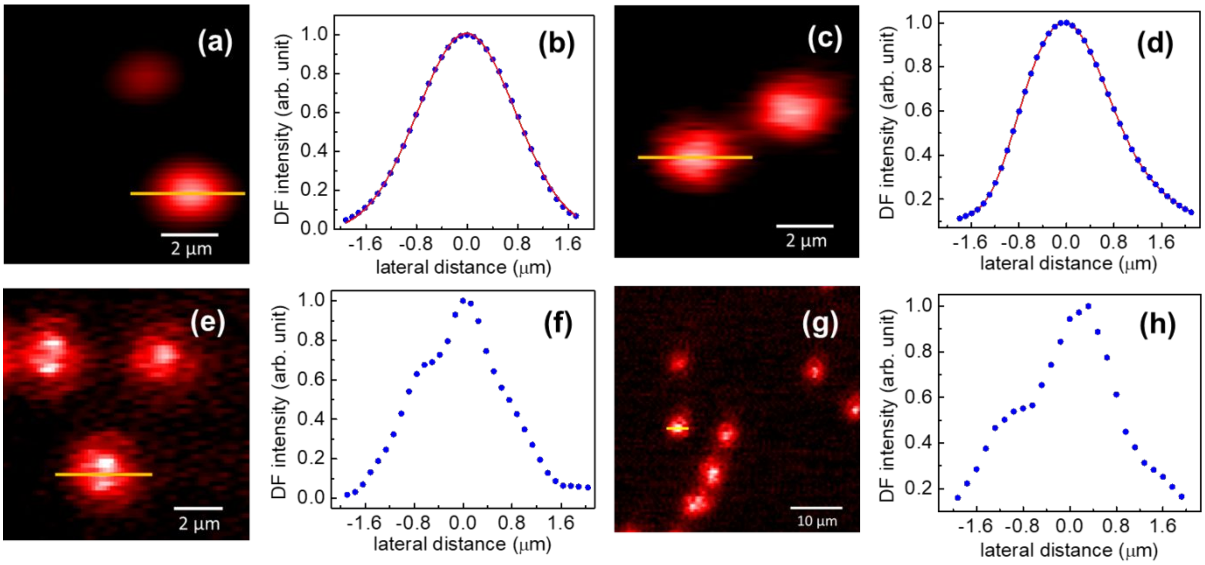

Figure S6. (a) High collection angle DF image of n-SiO_2 microparticles recorded with 633 nm CW laser (linear polarization). (b) Line profile extracted from (a). The red line is a Gaussian fit. (c) Same as (a) with OPA at 680 nm. (d) Line profile extracted from (c). The red line is a Gaussian fit. (e) High collection angle DF image of n-SiO_2@Ag microparticles recorded with OPA at 680 nm. (f) Line profile extracted from (e). (g) High collection angle DF image of n-SiO_2@EA/Ag microparticles recorded with OPA at 680 nm. (h) Line profile extracted from (g).

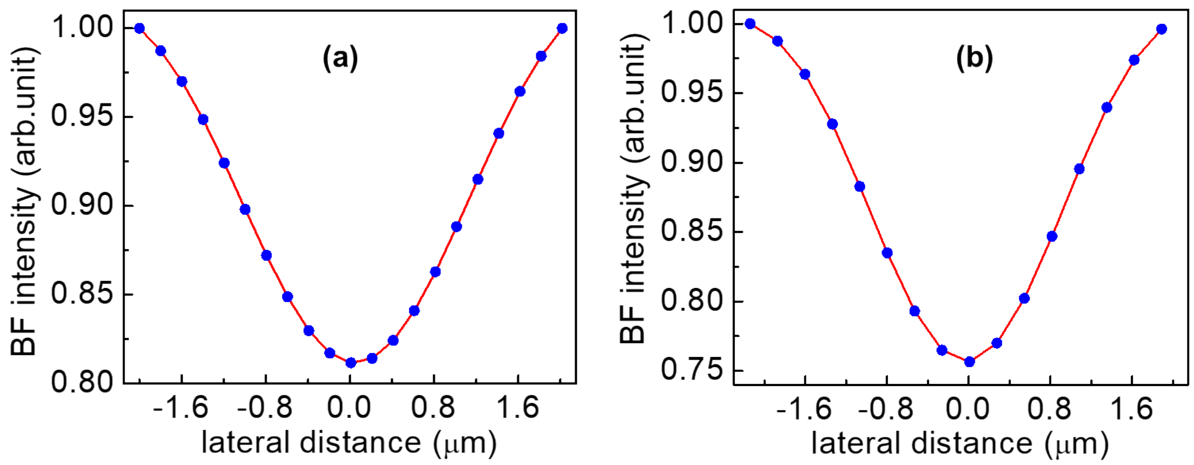

Figure S7. (a) Line profiles extracted from BF images of n-SiO_2@Ag microparticles with 633 nm laser. (b) Same with OPA tuned at 680 nm. Gaussian fits are shown in red. We used respectively 30 and 44 profiles to construct the histograms presented in Figure 3(l) and Figure 3(m).

**sample description**	**relevant dimension (nm)**	**dipole band wavelength (nm)**	**quadrupole band wavelength (nm)**	**reference**
Au sphere	240<br>340<br>560	800<br>900<br>>1000	610<br>630<br>810	1
Au nano-crescent	356	1284	962	2
Au dimer		715	650	3
Au nano-prism	144	1296	814	4
Au triangle	200	677	400	5
Ag sphere	113<br>189	496<br>715	413<br>567	6

Table S1. List of dipole and quadrupole band wavelengths reported in the literature for various nanoparticles. The quadrupole wavelength is in average 25% shorter than the dipole one.

References:

1. Chen, J.-D.; Xiang, J.; Jiang, S.; Dai, Q.-F.; Tie, S.-L.; Lan, S., Radiation of the high-order plasmonic modes of large gold nanospheres excited by surface plasmon polaritons. *Nanoscale* **2018,** *10* (19), 9153-9163.
2. Cooper, C. T.; Rodriguez, M.; Blair, S.; Shumaker-Parry, J. S. Polarization Anisotropy of Multiple Localized Plasmon Resonance Modes in Noble Metal Nanocrescents. *J. Phys. Chem. C* **2014**, *118*, 1167– 1173.
3. Theiss, J.; Aykol, M.; Pavaskar, P.; Cronin, S. B. Plasmonic Mode Mixing in Nanoparticle Dimers with Nm-Separations via Substrate-Mediated Coupling. *Nano Res.* **2014**, *7* (9) 1344– 1354.
4. Millstone, J. E.; Park, S.; Shuford, K. L.; Qin, L.; Schatz, G. C.; Mirkin, C. A.; Observation of a Quadrupole Plasmon Mode for a Colloidal Solution of Gold Nanoprisms. *J. Am. Chem. Soc.* **2005,** *127*, 5312 5313.
5. Chaturvedi, P.; Hsu, K. H.; Kumar, A.; Fung, K. H.; Mabon, J. C.; Fang, N. X. Imaging of Plasmonic Modes of Silver Nanoparticles Using High-Resolution Cathodoluminescence Spectroscopy. *ACS Nano* **2009**, *3* (10), 2965-2974.
6. Bastús, N. G.; Piella, J.; Puntes, V. Quantifying the Sensitivity of Multipolar (Dipolar, Quadrupolar, and Octapolar) Surface Plasmon Resonances in Silver Nanoparticles: The Effect of Size, Composition, and Surface Coating. *Langmuir* **2016**, *32* (1), 290-300.
